# Links between Metabolic Syndrome and Cardiovascular Autonomic Dysfunction

**DOI:** 10.1155/2012/615835

**Published:** 2012-03-15

**Authors:** G. Garruti, F. Giampetruzzi, M. G. Vita, F. Pellegrini, P. Lagioia, G. Stefanelli, A. Bellomo-Damato, F. Giorgino

**Affiliations:** ^1^Unit of Internal Medicine, Endocrinology, Andrology and Metabolic Diseases, Department of Emergency and Organ Transplantations (D.E.T.O.), University of Bari “A. Moro”, 70124 Bari, Italy; ^2^Department of Clinical Pharmacology and Epidemiology, Consorzio Mario Negri Sud, 66030 Santa Maria Imbaro, Chieti, Italy; ^3^Unit of Biostatistics, Scientific Institute Casa Sollievo della Sofferenza, San Giovanni Rotondo, 71013 Foggia, Italy

## Abstract

*Background*. Type 2 diabetes (T2D) might occur within metabolic syndrome (MbS). One of the complications of T2D is an impaired (imp) cardiovascular autonomic function (CAF). *Aims*. In subjects with T2D and age ≤ 55 years, the prevalence of impCAF and its relationship with BMI, waist, HbA_1c_ values, MbS, hypertension, and family history of T2D and/or hypertension were analysed. *Methods*. 180 subjects consecutively undergoing a day hospital for T2D were studied. The IDF criteria were used to diagnose MbS. To detect impCAF, 5 tests for the evaluation of CAF were performed with Cardionomic (Meteda, Italy). Univariate and multivariate analyses were performed. *Results*. The prevalence of impCAF and MbS were 33.9% and 67.8%, respectively. Among diabetics with impCAF, 86.9% had MbS. ImpCAF was significantly associated with MbS, overweight, and HbA_1c_ > 7%. Both logistic (*P* = 0.0009) and Poisson (*P* = 0.0113) models showed a positive association between impCAF and MbS. The degree of ImpCAF showed a positive linear correlation with BMI and HbA_1c_ values. *Conclusions*. The study demonstrates that glycaemic control and overweight influence CAF and that T2D + MbS is more strongly associated with impCAF than isolated T2D. We suggest that MbS not only increases the cardiovascular risk of relatively young subjects with T2D but is also associated with impCAF.

## 1. Introduction

Epidemiological studies demonstrated that diabetics display a cardiovascular risk which is twice that of sex- and age-matched nondiabetic population. In line with the high cardiovascular risk of subjects with diabetes mellitus (DM) are their frequent silent myocardial infarctions (MIs) [1, 2]. Clinically unrecognized MIs might be due to impaired cardiovascular autonomic function (impCAF) which finally evolves to cardiovascular autonomic neuropathy (CAN), a chronic complication of both type 1 and type 2 DM. In the Rochester diabetic neuropathy study concerning subjects with T2D, no correlation was found between autonomic symptoms and autonomic cardiovascular tests [3]. Therefore, an analysis of cardiovascular reflexes with tests which are sensitive and noninvasive allows to suspect diabetic CAN.

In subjects with DM, cardiovascular risk is known to be higher when clinical features of the metabolic syndrome (MbS) are present along with DM [4]. Several reports show that a higher cardiovascular risk is present in subjects displaying a cluster of factors predisposing to the atherosclerotic cardiovascular disease and included in the syndrome named MbS (Table 1) [5–7]. Subjects with T2D always have one of the diagnostic criteria of MbS (glycaemia ≥ 110 mg/dL), but do not obligatorily show other diagnostic features for MbS. In the present study we tried to assess whether MbS is more frequently associated with ImpCAF in relatively young type 2 diabetics.

## 2. Aims

Our study evaluated the association, if any, between an early deficit of CAF and the presence of MbS defined on the criteria of the International Diabetes Federation (IDF) [6] and whether any correlation existed between the detection of an early deficit of CAF and HbA_1c_, the duration of T2D and/or hypertension (HBP), the occurrence of a positive family history of DM, HBP, or both, and the nutritional habits in a cohort of type 2 diabetics not older than 55 years.

## 3. Methods

The study included subjects with T2D and age ≤ 55 years consecutively undergoing a day hospital (DH) for chronic complications of DM at the Unit of Endocrinology of the University Hospital of Bari from October 2004 to September 2006. We screened 210 subjects. Thirty subjects out of 210 were excluded because they could not be screened for cardiovascular reflexes (11 experienced acute MI less than 6 months before DH and the remaining 19 showed arrhythmias at the basal ECG at DH admission). 180 type 2 diabetics (117 males and 63 females) with mean age of 48.62 ± 6.12 years (48.18 ± 7.26 and 48.86 ± 5.46 for female and males, resp.) were recruited and underwent 5 different tests for cardiovascular reflexes. At DH admission, all subjects gave their written informed consent. The tests included beat-to-beat heart rate variation (DB), heart rate response to standing (lying to standing, LS), heart rate response to Valsalva maneuver (Vs), heart rate response to cough (cough test, CT), and systolic blood pressure response to standing (PH) [1, 8–11]. All tests were performed with Cardionomic [8, 9], which is a portable computerised system that is used for step-by-step performance of several cardiovascular tests for autonomic neuropathy. All tests were performed after an overnight fast but never after overnight hypoglycaemia. Each subject was instructed to refrain from smoking and drinking coffee at least 8 h before tests. Before the tests, patients were lying in the supine position for 30 minutes and a basal ECG was performed. As far as DB is concerned, it evaluates the physiologic arrhythmia induced by respiration and is an index of the vagus nerve function. Inspiration induces pulmonary expansion which stimulates stretch receptors in the lungs, in the atrium, and in the chest wall. The above-mentioned receptors stimulate the nucleus solitarius and the bulbar cardioinhibitory center through afferent vagal fibers. The final effect is the inhibition of the vagus which is followed by the heart rate increase. During expiration, opposite mechanisms occur which induce heart rate deceleration. Therefore, respiratory arrhythmia is mainly due to the prevailing effect of the parasympathetic nervous system. When parasympathetic autonomic dysfunction occurs, the respiration-induced heart rate variation is decreased or abolished.

For DB, a parasympathetic test function, a 1 min ECG was performed when the subject was lying supine and deeply breathed 6 times per minute. The expiration/inspiration R-R ratio was calculated. For LS, a parasympathetic test function, the patient was invited to stand suddenly and the R-R interval was measured at beats 15 and 30 after standing and the 30/15 ratio was calculated. 

VS simultaneously evaluates parasympathetic, sympathetic, and baro receptor functions. For VS, the patient exhales for 15 min into the mouthpiece of a manometer exerting a pressure of 40 mmHg. The ratio of longest-to-shortest R-R interval was measured. For HP assessment, supine systolic blood pressure was measured after the patient was lying down for 30 min and orthostatic blood pressure after the patient was standing for 2 minutes. Orthostatic hypotension was diagnosed when the fall in systolic blood pressure (SBP) levels was ≥30 mmHg or that of diastolic BP (DBP) was >10 mmHg in response to a postural change from supine to upright position [12]. Orthostatic hypotension is known to reflect sympathetic dysfunction [13]. CT, a parasympathetic test function, evaluates the cough-mediated increase in heart rate. During the test, the patient was in the supine position and ECG was performed when patient breathed for 15 seconds (basal) and again when he coughed 3 times. The R-R ratio between the shortest R-R interval after the last cough and the mean R-R interval during regular respiration was calculated [10, 11].

Since for each test the range of normal values was changing with age, we elaborated a score grading from 0 (normal response to all performed tests) to 5 (impaired response to all performed tests). Normal values for tests were according to Vespasiani et al. [8] but were also confirmed in a cohort of age- and sex-matched control subjects selected in our region (*n* = 130). Part of this cohort of controls was already used to validate CAF tests in a cohort of subjects with *β*-thalassemia [9]. Control subjects showed normal glucose tolerance (normal fasting glucose circulating levels and HbA_1c_ levels < 5.9%), they did not display any of the diagnostic features of the MbS, and they were not taking any pharmacological treatment. 

We also analysed whether any relationship existed between the detection of different degrees of impCAF and the presence of MbS according to the International Diabetes Federation [6]. The nutritional habits were also assessed by a dietician through a dietary interview. HbA_1c_ was measured in HPLC (Menarini Diagnostics). Total cholesterol, HDL cholesterol, LDL cholesterol, and triglycerides circulating levels were measured with specific Dimension clinical chemistry systems (Siemens Healthcare Diagnostics Ltd.) which are *in vitro* tests intended for the quantitative determination in human serum or plasma. 

### 3.1. Statistical Analysis

Data are expressed as mean ± SD or %. Two-sided *P* values refer to the Mann-Whitney *U* test for continuous variables and Pearson's *χ* ^2^ for categorical variables. Univariate and multivariate analyses for at least one positive CAF test versus none and mean number of positive CAF tests were, respectively, assessed with logistic and Poisson's regression models. Results are expressed as odds ratios (ORs) or rate ratios (RRs) and their 95% confidence intervals (CIs). Two-sided *P* values less than 0.05 were considered significant. All the analyses were performed using SAS (Release 9.1, SAS Institute, Cary, NC, USA, 2002-2003). 

## 4. Results

### 4.1. Prevalence of Impaired CAF

**Figure 1:**
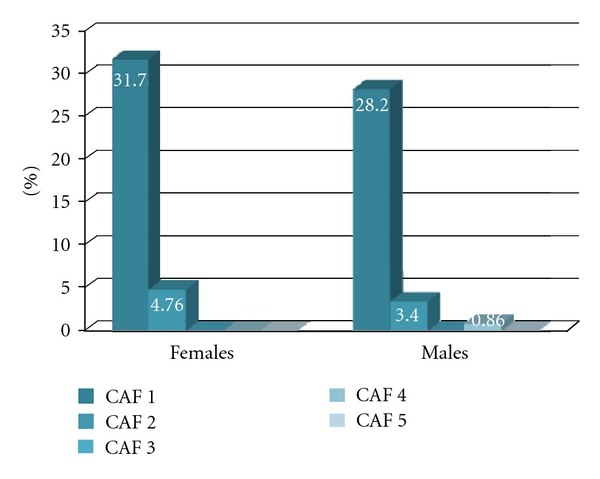
Distribution of different scores of impaired CAF in the cohort.

Patients' characteristics of the sample by number of positive tests for impaired cardiovascular autonomic function (CAF) are reported in Tables 2(a), 2(b), and 2(c). In our cohort 33.9% subjects (61 out of 180) showed at least one pathologic test for CAF. Among female patients, 4.76% showed a pathological response to DB, 20.6% and 9.5% showed pathological responses to Vs and CT, respectively. Among male patients, 4.3% showed a pathological response to DB, 6.84% to LS, 18% to VS, and 6.84% to CT. Among females, no subject showed a score ranging from 3 to 5. Score 2 was found in 4.76% and score 1 in 31.7% of female subjects (Figure 1). Among male subjects, nobody showed score 3 or 5 and less than 1% (0.86%) had a score 4. The distribution of scores 1 and 2 was comparable to that found in the female cohort since 28.2% and 3.4% of male subjects showed scores 1 and 2, respectively (Figure 1).

### 4.2. Impaired CAF and Anthropometric and Metabolic Variables

**Figure 2:**
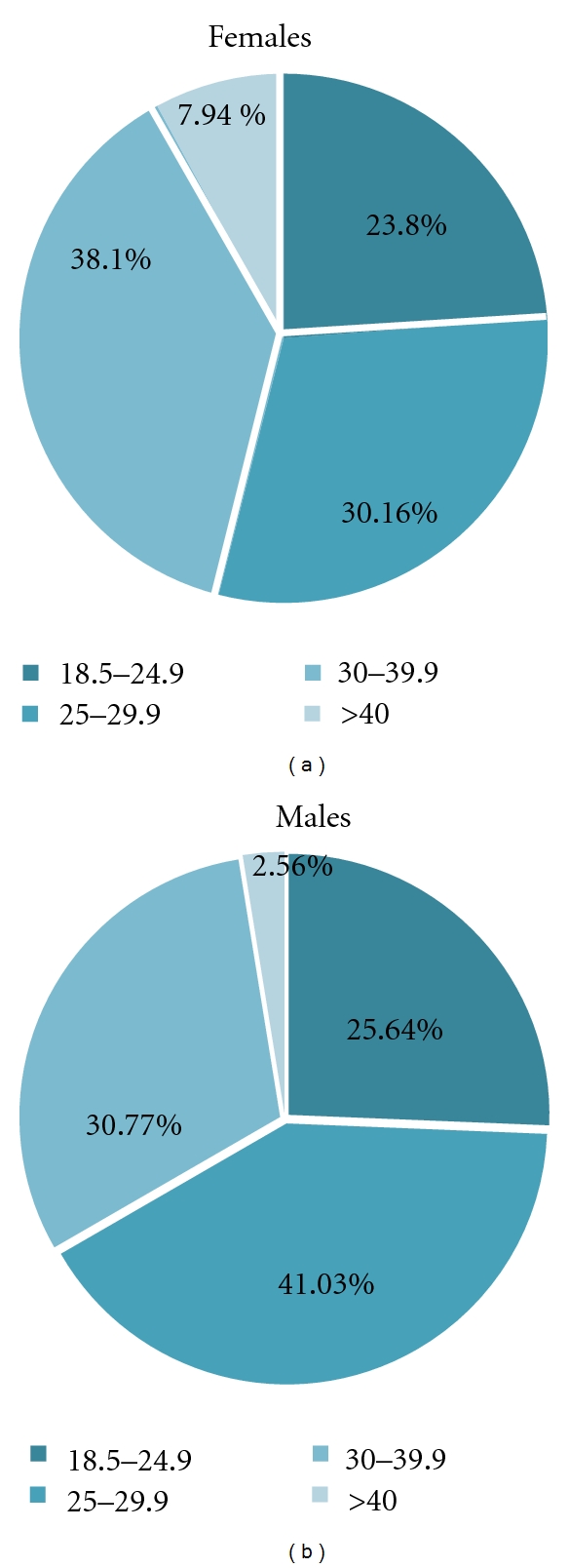
Distribution of BMI classes in the cohort.

**Figure 3:**
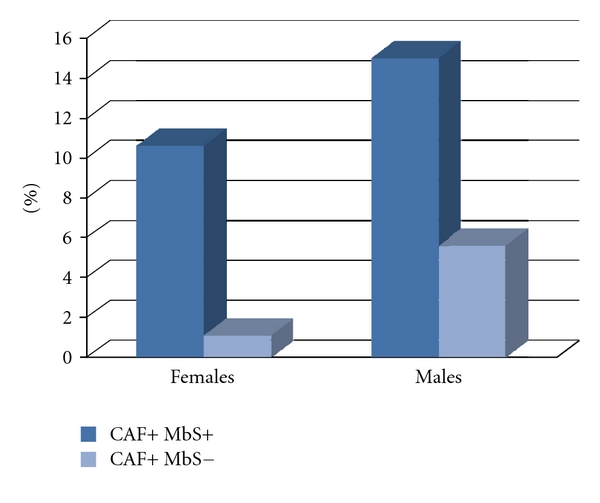
Prevalence of metabolic syndrome in the cohort in the presence or in the absence of impaired CAF. Abbreviations: CAF+, presence of impaired CAF; MbS+, presence of Metabolic syndrome; MbS−, absence of metabolic syndrome. Notes. Sixty patients out of 180 had an impaired CAF.

**Figure 4:**
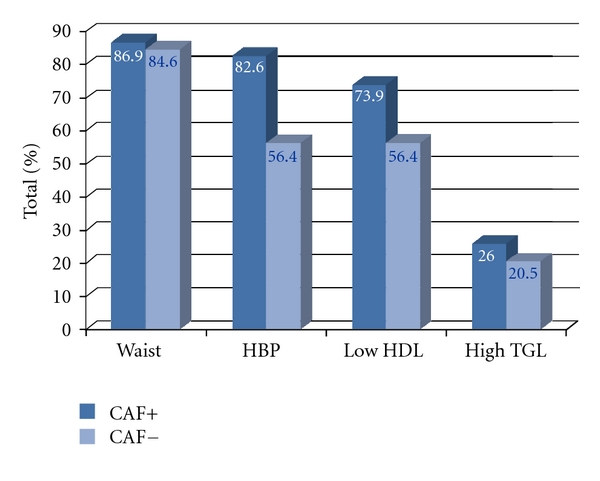
Distribution of different components of the metabolic syndrome in the female cohort in the presence or in the absence of impaired CAF. Abbreviations: CAF+: presence of impaired CAF; CAF−: absence of impaired CAF.

**Figure 5:**
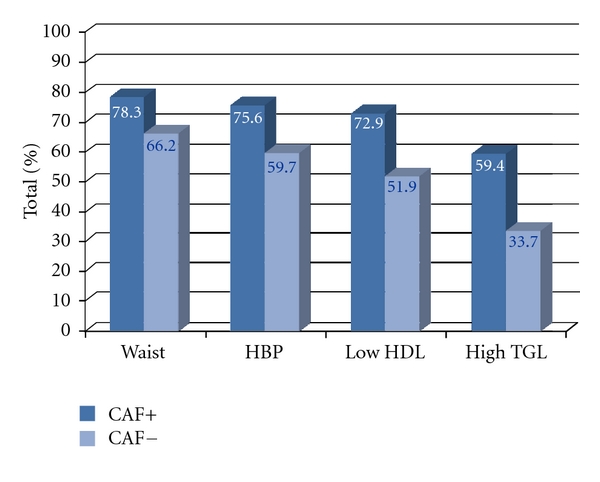
Distribution of different components of the metabolic syndrome in the male cohort in the presence or in the absence of impaired CAF. Abbreviations: CAF+: presence of impaired CAF; CAF−: absence of impaired CAF.

When female diabetics were stratified for BMI classes, we found 23.8% normal-weight, 30.16% overweight, 38.1% obese (class 1 and 2), and 7.94% severe obese women (BMI ≥ 40 Kg/m^2^) (Figure 2). Among male subjects, 25.64% were normal-weight, 41.03% overweight, 30.77% obese (class 1 and 2), and 2.56% severely obese (BMI ≥ 40 Kg/m^2^) (Figure 2). The prevalence of MbS in the presence of impCAF was significantly higher than that in the absence of impCAF in both sexes (Figure 3). The distribution of the different components of the MbS in the male and female cohort in the presence or in the absence of impCAF was similar (Figures 4 and 5). 

When subjects were stratified for both CAF score and BMI classes, the presence of at least one pathologic test for CAF showed a significant positive correlation with BMI > 25 Kg/m^2^ (*P* = 0.0227, Table 3). Subjects with at least one pathologic test had BMI (*P* = 0.0032), waist circumferences (*P* = 0.0146), triglycerides (*P* = 0.0089), and HbA_1c_ (*P* = 0.0292) levels significantly higher than those of subjects with normal tests. The occurrence either of one or more abnormal tests was not significantly associated with a positive FH for DM and/or HBP or with a duration of T2D longer than 5 years. By contrast, the occurrence of at least one pathological test was positively associated to the occurrence of HBP (*P* = 0.0061), a waist value >94 cm (according to IDF) (*P* = 0.0146), and triglycerides ≥150 (*P* = 0.0089). In the multivariate Poisson model, we considered the association between the mean number of tests positive for impaired CAF and age, sex, classes of BMI, FH of DM, and duration of DM longer than 5 years (Table 4). A statistically significant association with the mean number of positive tests for impaired CAF was found when MbS was considered as dichotomous (*P* = 0.0018). Significant associations were also found between the mean number of tests positive for impaired CAF and the occurrence of overweight (BMI between 25 and 30 Kg/m^2^) and HbA_1c_ > 7% (Table 4). With adjustment for BMI classes, FH of diabetes, and/or hypertension, there was still a significant association between the mean number of tests positive for impaired CAF and HbA_1c_ > 7% (Table 4). With additional adjustment for sex, significant associations of the mean number of tests positive for impaired CAF with HbA_1c_ > 7% and the occurrence of MbS were confirmed (Table 4). 

### 4.3. Impaired CAF, MbS, and Nutrient Intake

Significant associations were found between the mean number of tests positive for impCAF and a lipid intake >30% (Table 3). 

When patients were stratified according to a daily lipid intake >30%, the occurrence of at least one pathologic CAF test was significantly associated to a lipid intake >30% (41.7% at least one versus 19.6% none *P* = 0.0048) (Table 3). 

In a univariate analysis, a significant correlation was found between the mean number of tests positive for impaired CAF and a protein intake <15% and a lipid intake >30%. However, in the multivariate analysis protein and lipid content does not predict impCAF (Table 4). 

## 5. Discussion

The study of both micro- and macroangiopathic complications of DM is crucial for both prognosis and therapeutic strategy. Among chronic microangiopathic complications of DM, CAN involves the cardiovascular branch of the autonomic nervous system (ANS) [14–17]. Because of CAN, diabetics might experience silent MI, silent hypoglycaemia, and a high ASA risk during major surgery. ANS is anatomically poorly accessible, and few direct physiological tests are available to study CAF. Therefore, some indirect clinical tests are used as screening tests which detect deficits of CAF on the basis of heart responses to a simple stimulus [18]. In subjects with pathologic screening tests, the diagnosis might be completed with more sensitive techniques, but indirect screening tests help to select candidate subjects for more sophisticated analyses [18]. The diagnosis of CAN is usually done when at least 2 screening tests display pathologivc responses [18], but often when more than one test is already impaired and the diagnosis of CAN is made is not possible to reverse the situation. Vice versa sometimes early parasympathetic neuropathy may improve. In a longitudinal study Gottsäter et al. [19] demonstrated that after 7–10 years some subjects with parasympathetic neuropathy did not fulfill the criteria for the diagnosis anymore. Therefore we thought to consider as an early deficit of CAF the detection of at least one pathological test (score 1). CAF was analysed by utilizing five different tests. To each pathologic test, we gave score 1 to establish a grading of severity of impCAF. In our cohort of relatively young subjects with T2D, a score of impCAF higher than 2 was luckily rare, but the prevalence of at least one pathologic test was 33.9%. In two multicenter studies and a population study of type 2 diabetics, the prevalence of CAN was 16–22% [16, 20, 21]. The prevalence we found was slightly higher. However, in the above-mentioned studies, 2 screening tests (DB, LS) or 3 (DB, LS, PH) were used. By contrast, in our small cohort, 5 tests were always performed in triplicate thus increasing the sensitivity of tests. Concerning MbS, in our young cohort, 65% subjects had MbS according to IDF, but the prevalence of MbS among the subjects showing at least one pathologic test of CAF was more than 85%. A significant positive correlation between impaired CAF and MbS was confirmed with two different models of multivariate analysis. It was previously assessed an association between parasympathetic dysfunction (pathologic cardiac response to DB) and some features of the MbS according to the WHO [22–24]. However, to our knowledge this is the first report stating that MbS, according to the criteria of IDF, is associated with a higher occurrence of an early deficit of CAF in a relatively young cohort of type 2 diabetics. In the same cohort, we also analysed the possible associations between the single components of MbS and the detection of an early deficit of CAF. However, score 1 was strongly associated with most of the components of the MbS. 

We found a significant correlation between the occurrence of at least one pathologic test of CAF and a BMI > 25, which supports the negative role played by overweight on cardiovascular risk. The link between the high cardiovascular risk of T2D and overweight might be explained considering the negative effect played by overweight on glycaemic control. In this line of evidence in our cohort, a significant association was found between high HbA_1c_ values and CAF score. When subjects were stratified on HbA_1c_ values higher or lower than 7, a significant association was found between HbA_1c_> 7 and the occurrence of at least one pathologic test. Many studies have already demonstrated that either an acute or a chronic poor glycaemic control might help the appearance of CAN [25–27]. From different meta-analyses the median value of mortality after 5 years was around 25% in diabetics with CAN and 4% in diabetics without CAN. If the diagnosis of CAN was based on the occurrence of 2 pathological tests, the relative risk of mortality was 3.5 [28, 29]. By contrast, an improvement in glycaemic control improves an early deficit of CAF or stops its progression [25]. In studies utilizing heart rate variability as an index of CAF, mild CAF abnormalities improved if HbA_1c_ values decreased from 9.5% to 8.4% [26]. 

Interestingly subjects showing impaired CAF also show different dietary habits as compared with subjects with normal CAF since they consume a higher daily fat intake (increased consumption of saturated fat derived from cheese and meet) as compared with diabetics with normal CAF. In some reports it has already been stated [30] that in subjects with MbS a Mediterranean-style diet (high content of whole grains, fruits, vegetables, nuts, and olive oil) improves chronic low-grade inflammatory state (reduction in serum concentrations of C-reactive protein, interleukin 6, insulin resistance, and improved endothelial function score) as compared to a balanced low-fat diet. Our data suggest that subjects with impaired CAF and MbS chose a wrong diet even if they were living in a Mediterranean area. 

Unexpectedly no association was found between CAF score and the duration of diabetes or a positive family history of DM and/or HBP. In other papers a strong association was found between the duration of diabetes and CAN [23]. In several studies both PH and decreased heart rate variability are more frequent and evident 5 years after the diagnosis of Diabetes [23]. However, the subjects of our cohort were younger than those considered in previous studies and they experienced a program of education to healthy life style together with drugs (glitazone or insulin analogues) of last generation since the onset of diabetes, thus showing a metabolic memory better than that of subjects from previous studies. 

The lack of association between any deficit of CAF and a positive family history of DM probably suggest that genetic and familiar factors might play a minor influence in compromising CAF as compared with environmental factors such as glycaemic control. 

In conclusion, our data strongly suggest the role played by glycaemic control (assessed on the basis of HbA_1c_ values) and overweight on an early deficit of CAF. The more significant association between MbS and impaired CAF as compared with isolated T2D might suggest that the presence of MbS not only increases the global cardiovascular risk of diabetics not older than 55 years but also accelerates the appearance of a deficit of CAF which additionally increase cardiovascular risk. 

## Figures and Tables

**Table 1:** Diagnostic criteria for the metabolic syndrome.

Any 3 of the following conditions		
*(1) Central obesity*		

According to NCEPIII*		

Country/ethnic group	Sex	Waist in cm

Any	Male	≥102 cm
	Female	≥88 cm

Or according to IDF^†^		

Country/ethnic group	Sex	Waist in cm

Europids	Male	≥94
	Female	≥80
South Asians, Chinese	Male	≥90
	Female	≥80
Japanese	Male	≥85
	Female	≥90

(2) Elevated triglyceridemia (≥150 mg/dL).		
(3) Decreased HDL cholesterolemia (<40 mg/dL in males, <50 mg/ dL in females).		
(4) Elevated arterial blood pressure (≥130/85 mmHg).		
(5) Elevated fasting blood glucose (≥110 mg/dL or ≥100 mg/dL according to IDF^†^).		

*Adapted from the third report of the National Cholesterol Education Program (NCEPIII) Expert Panel on Detection, Evaluation, and Treatment of High Blood Cholesterol in Adults (Adult Treatment Panel III, ATP III). ^†^International Diabetes Federetion.

**Table:** (a)

Variable	Category	Number of positive CAF tests		All	*P* value
		At least one	None		
*N*		61	119	180	

Age		48.9 ± 5.3	48.5 ± 6.5	48.6 ± 6.1	0.9372

BMI		31.6 ± 7.3	28.0 ± 5.0	29.2 ± 6.1	0.0032

BMI class	<25	10 (16.4)	35 (29.4)	45 (25.0)	0.0227
	25–30	21 (34.4)	49 (41.2)	70 (38.9)	
	≥30	30 (49.2)	35 (29.4)	65 (36.1)	

Duration		6.0 ± 5.9	6.4 ± 6.5	6.3 ± 6.3	0.9071

Duration ≥5 years	Not assessed	3 (·)	5 (·)	8 (·)	0.4557
	No	34 (58.6)	60 (52.6)	94 (54.7)	
	Yes	24 (41.4)	54 (47.4)	78 (45.3)	

Family diabetes	No	11 (18.0)	23 (19.3)	34 (18.9)	0.8336
	Yes	50 (82.0)	96 (80.7)	146 (81.1)	

Family hypertension	No	38 (62.3)	71 (59.7)	109 (60.6)	0.7324
	Yes	23 (37.7)	48 (40.3)	71 (39.4)	

Fibers		27.6 ± 4.2	27.8 ± 4.0	27.7 ± 4.1	0.9747

HDL-cholesterol		39.1 ± 10.1	42.5 ± 11.3	41.3 ± 11.0	0.0685

HbA_1c_		7.7 ± 1.4	7.3 ± 1.5	7.4 ± 1.5	0.0292

HbA_1c_> 6.5%	Not assessed	1 (·)	0 (·)	1 (·)	0.0864
	No	15 (25.0)	45 (37.8)	60 (33.5)	
	Yes	45 (75.0)	74 (62.2)	119 (66.5)	

HbA_1c_> 7%	Not assessed	1 (·)	0 (·)	1 (·)	0.0299
	No	25 (41.7)	70 (58.8)	95 (53.1)	
	Yes	35 (58.3)	49 (41.2)	84 (46.9)	

Height		1.6 ± 0.1	1.7 ± 0.1	1.7 ± 0.1	0.0429

Fibers >30 g/diet	Not assessed	13 (·)	22 (·)	35 (·)	0.8568
	No	26 (54.2)	51 (52.6)	77 (53.1)	
	Yes	22 (45.8)	46 (47.4)	68 (46.9)	

Hypertension	No	27 (44.3)	78 (65.5)	105 (58.3)	0.0061
	Yes	34 (55.7)	41 (34.5)	75 (41.7)	

Lipids >30%	Not assessed	13 (·)	22 (·)	35 (·)	0.0048
	No	28 (58.3)	78 (80.4)	106 (73.1)	
	Yes	20 (41.7)	19 (19.6)	39 (26.9)	

Metabolic syndrome	No	8 (13.1)	50 (42.0)	58 (32.2)	<0.0001
	Yes	53 (86.9)	69 (58.0)	122 (67.8)	

Data are expressed as mean ± SD or %. Two-sided *P* values refer to the Mann-Whitney *U* test for continuous variables and Pearson's *χ* ^2^ for categorical variables.

**Table:** (b)

Variable	Category	Number of positive CAF tests		All	*P* value
		At least one	None		

Metabolic syndrome score	1	3 (4.9)	13 (10.9)	16 (8.9)	0.0001
	2	5 (8.2)	37 (31.1)	42 (23.3)	
	3	18 (29.5)	27 (22.7)	45 (25.0)	
	4	23 (37.7)	34 (28.6)	57 (31.7)	
	5	12 (19.7)	8 (6.7)	20 (11.1)	

Proteins >15%	Not assessed	13 (·)	22 (·)	35 (·)	0.0838
	No	24 (50.0)	34 (35.1)	58 (40.0)	
	Yes	24 (50.0)	63 (64.9)	87 (60.0)	
Glucides >55%	Not assessed	13 (·)	22 (·)	35 (·)	0.4146
	No	6 (12.5)	8 (8.2)	14 (9.7)	
	Yes	42 (87.5)	89 (91.8)	131 (90.3)	

Sex	Female	23 (37.7)	40 (33.6)	63 (35.0)	0.5859
	Male	38 (62.3)	79 (66.4)	117 (65.0)	

Triglycerides		167.3 ± 92.1	140.9 ± 95.8	149.9 ± 95.1	0.0089

Waist circumference		104.9 ± 17.9	97.8 ± 12.6	100.2 ± 15.0	0.0146

Weight		85.0 ± 21.3	78.1 ± 14.7	80.4 ± 17.5	0.0597

Data are expressed as mean ± SD or %. Two-sided *P* values refer to the Mann-Whitney *U* test for continuous variables and Pearson's *χ* ^2^ for categorical variables.

**Table:** (c)

Antidiabetic therapy	Sex	Patients/total
Diet alone	Male	34/117
	Female	17/63
Diet + metformin	Male	26/117
	Female	25/63
Diet + sulphonylureas or glinides	Male	15/117
	Female	3/63
Diet + metformin+	Male	27/117
sulphonylureas or glinides	Female	8/63
Diet + insulin (basal/bolus)	Male	6/117
	Female	6/63

Notes. (1) The remaining 4 female subjects out of 63 were treated either with Insulin (basal/bolus) plus metformin or with insulin (basal/bolus) plus sulphonylureas or glinides plus metformin).

(2) The remaining 9 male subjects out of 117 were treated either with insulin basal plus glitazones and metformin or with insulin basal plus sulphonylurea and metformin.

**Table 3:** Association between impaired CAF and anthropomentric and metabolic variables.

BMI ≥ 24.9 Kg/m^2^	*P* = 0,0032
Hypertension	*P* = 0,0061
Waist > 94/80 cm	*P* = 0,0146
Tryglycerides > 150 mg/dL	*P* = 0,0089
HbA_1c_≥ 7%	*P* = 0,0299
Protein intake ≤15%/day	*P* = 0,0838
Lipid intake > 30%	*P* = 0,0048
Metabolic syndrome	*P* < 0,0001

BMI: Body mass index was calculated as Kg/m^2^.

**Table 4:** 

	Variable	Age	BMI class			Family DM2	Duration > 5 yrs	Fibers > 30 g/die	Glucides 55%	HbA_1c_ > 7%	Family hypertension	Lipids > 30%	Metabolic syndrome	Proteins > 15%	Sex
	Category		*<25*	*25–30*	*≥30*										

Univariate	IRR	0.99	0.68	0.51	1.00	1.14	0.79	1.02	0.87	1.91	0.89	2.10	2.90	0.60	0.93
	IRR 95% CI	0.96–1.03	0.41–1.14	0.26–0.98	1.00-1.00	0.62–2.13	0.49–1.29	0.60–1.72	0.37–2.04	1.18–3.11	0.55–1.44	1.23–3.59	1.49–5.66	0.35–1.01	0.58–1.51
	*P* value	0.7451	0.1475	**0.0448**	—	0.669	0.351	0.9554	0.7531	**0.0087**	0.6265	**0.0062**	**0.0018**	***0.0567***	0.7748

Multivariate	IRR	0.98	1.45	2.32	1.00	1.18	0.71	1.44	0.71	2.02	0.92	1.93	2.53	0.65	0.93
	IRR 95% CI	0.93–1.04	0.69–3.02	0.82–6.53	1.00-1.00	0.53–2.64	0.39–1.29	0.76–2.72	0.25–2.03	1.09–3.73	0.52–1.63	0.82–4.50	1.08–5.90	0.30–1.39	0.51–1.69
	*P* value	0.4908	0.3265	0.1115	—	0.683	0.2609	0.2682	0.5197	**0.0256**	0.7664	0.13	**0.0319**	0.2649	0.802

Multivariate	IRR	0.98	1.4	2.45	1.00		0.7			1.92		1.97	2.50	0.76	1.00
	IRR 95% CI	0.94–1.03	0.68–2.91	0.88–6.85	1.00-1.00		0.39–1.25			1.07–3.46		0.87–4.47	1.08–5.78	0.38–1.52	0.55–1.80
	*P* value	0.5131	0.3614	***0.0863***	—		0.2241			**0.0297**		0.106	**0.0318**	0.4357	0.9944

Multivariate	IRR	0.98					0.72			1.95		1.47	1.88	0.85	1.08
	IRR 95% CI	0.94–1.03					0.40–1.29			1.08–3.53		0.73–2.93	0.90–3.96	0.43–1.66	0.60–1.92
	*P* value	0.5243					0.263			**0.0268**		0.2775	***0.0948***	0.6309	0.8003

Multivariate	IRR	0.98					0.72			1.96		1.62	1.90		1.06
	IRR 95% CI	0.94–1.03					0.40–1.30			1.08–3.54		0.92–2.85	0.90–3.99		0.60–1.89
	*P* value	0.5333					0.2797			**0.0261**		***0.097***	***0.0909***		0.8385

Multivariate	IRR	0.98								1.77		1.60	2.08		0.97
	IRR 95% CI	0.93–1.03								0.99–3.15		0.91–2.80	0.99–4.34		0.55–1.70
	*P* value	0.3911								***0.0529***		0.1021	***0.052***		0.9098

Multivariate	IRR	0.98								1.97			2.91		0.93
	IRR 95% CI	0.94–1.02								1.20–3.24			1.48–5.71		0.57–1.52
	*P* value	0.3205								**0.0075**			**0.0019**		0.7838

Multivariate	IRR	0.98								1.96			2.91		
	IRR 95% CI	0.94–1.02								1.19–3.22			1.48–5.71		
	*P* value	0.3223								**0.0078**			**0.0019**		

Multivariate	IRR									1.87			2.82		
	IRR 95% CI									1.15–3.04			1.44–5.51		
	*P* value									**0.0113**			**0.0024**		

Statistical analysis was performed with Poisson's model.

## References

[1] Vinik AI, Erbas T (2001). Recognizing and treating diabetic autonomic neuropathy. *Cleveland Clinic Journal of Medicine*.

[2] Langer A, Freeman MR, Josse RG, Steiner G, Armstrong PW (1991). Detection of silent myocardial ischemia in diabetes mellitus. *American Journal of Cardiology*.

[3] Low PA, Benrud-Larson LM, Sletten DM (2004). Autonomic symptoms and diabetic neuropathy: a population-based study. *Diabetes Care*.

[4] Church TS, Thompson AM, Katzmarzyk PT (2009). Metabolic syndrome and diabetes, alone and in combination, as predictors of cardiovascular disease mortality among men. *Diabetes Care*.

[5] Grundy SM, Cleeman JI, Daniels SR (2005). Diagnosis and management of the metabolic syndrome: an American heart association/national heart, lung, and blood institute scientific statement. *Circulation*.

[7] Zimmet P, Magliano D, Matsuzawa Y, Alberti G, Shaw J (2005). The metabolic syndrome: a global public health problem and a new definition. *Journal of atherosclerosis and thrombosis.*.

[8] Vespasiani G, Bruni M, Meloncelli I (1996). Validation of a computerised measurement system for guided routine evaluation of cardiovascular autonomic neuropathy. *Computer Methods and Programs in Biomedicine*.

[9] Portincasa P, Moschetta A, Berardino M (2004). Impaired gallbladder motility and delayed orocecal transit contribute to pigment gallstone and biliary sludge formation in *β*-thalassemia major adults. *World Journal of Gastroenterology*.

[10] Cardone C, Bellavere F, Ferri M, Fedele D (1987). Autonomic mechanisms in the heart rate response to coughing. *Clinical Science*.

[11] Cardone C, Paiusco P, Marchetti G, Burelli F, Feruglio M, Fedele D (1990). Cough test to assess cardiovascular autonomic reflexes in diabetes. *Diabetes Care*.

[12] Freeman R, Landsberg L, Young J (1999). The treatment of neurogenic orthostatic hypotension with 3,4-DL-threo- dihydroxyphenylserine: a randomized, placebo-controlled, crossover trial. *Neurology*.

[13] Low PA, Walsh JC, Huang CY, McLeod JG (1975). The sympathetic nervous system in diabetic neuropathy: a clinical and pathological study. *Brain*.

[14] Appenzeller O (1991). Report on the 20^th^ International Congress of Neurovegetative (Autonomic) Research, September 1990, Tokyo, Japan. *Clinical Autonomic Research*.

[15] Ravits JM (1997). AAEM minimonograph 48: autonomic nervous system testing. *Muscle and Nerve*.

[16] Ziegler D, Gries FA, Muhlen H (1993). Prevalence and clinical correlates of cardiovascular autonomic and peripheral diabetic neuropathy in patients attending diabetes centers. *Diabete et Metabolisme*.

[17] Vinik AI, Ziegler D (2007). Diabetic cardiovascular autonomic neuropathy. *Circulation*.

[18] Freeman R (2006). Assessment of cardiovascular autonomic function. *Clinical Neurophysiology*.

[19] Gottsäter A, Kangro M, Sundkvist G (2004). Do high proinsulin and C-peptide levels play a role in autonomic nervous dysfunction? Power spectral analysis in patients with non-insulin-dependent diabetes and nondiabetic subjects. *Diabetic Medicine*.

[20] Stephenson J, Fuller JH (1994). Microvascular and acute complications in IDDM patients: the EURODIAB IDDM Complications Study. *Diabetologia*.

[21] Neil HAW, Thompson AV, John S, McCarthy ST, Mann JI (1989). Diabetic autonomic neuropathy: the prevalence of impaired heart rate variability in a geographically defined population. *Diabetic Medicine*.

[22] Gottsäter A, Ahmed M, Fernlund P, Sundkvist G (1999). Autonomic neuropathy in Type 2 diabetic patients is associated with hyperinsulinaemia and hypertriglyceridaemia. *Diabetic Medicine*.

[23] Szelag B, Wroblewski M, Castenfors J (1999). Obesity, microalbuminuria, hyperinsulinemia, and increased plasminogen activator inhibitor 1 activity associated with parasympathetic neuropathy in type 2 diabetes. *Diabetes Care*.

[24] Takayama S, Sakura H, Katsumori K, Wasada T, Iwamoto Y (2001). A possible involvement of parasympathetic neuropathy on insulin resistance in patients with type 2 diabeties. *Diabetes Care*.

[25] DCCT Research Group (1993). The effect of intensive treatment of diabetes on the development and progression of long-term complications in insulin-dependent diabetes mellitus. *New England Journal of Medicine*.

[26] Burger AJ, Weinrauch LA, D’Elia JA, Aronson D (1999). Effect of glycemic control on heart rate variability in type I diabetic patients with cardiac autonomic neuropathy. *American Journal of Cardiology*.

[27] Gaede P, Vedel P, Parving HH, Pedersen O (1999). Intensified multifactorial intervention in patients with type 2 diabetes mellitus and microalbuminuria: the Steno type 2 randomised study. *The Lancet*.

[28] Shaw JE, Zimmet PZ, Gries FZ, Ziegler D, Griesw FA, Cameron NE, Low PA, Ziegler D (2003). Epidemiology of diabetic neuropathy. *Textbook of Diabetic Neuropathy*.

[29] Vinik AI, Maser RE, Mitchell BD, Freeman R (2003). Diabetic autonomic neuropathy. *Diabetes Care*.

[30] Esposito K, Marfella R, Ciotola M (2004). Effect of a mediterranean-style diet on endothelial dysfunction and markers of vascular inflammation in the metabolic syndrome: a randomized trial. *Journal of the American Medical Association*.

